# Supplementary figures and images for: A molecular atlas of plastid and mitochondrial proteins reveals organellar remodeling during plant evolutionary transitions from algae to angiosperms
Source: PLoS Biol. 2024 May 7;22(5):e3002608. doi: 10.1371/journal.pbio.3002608 (PMC11135702; doi:10.1371/journal.pbio.3002608)

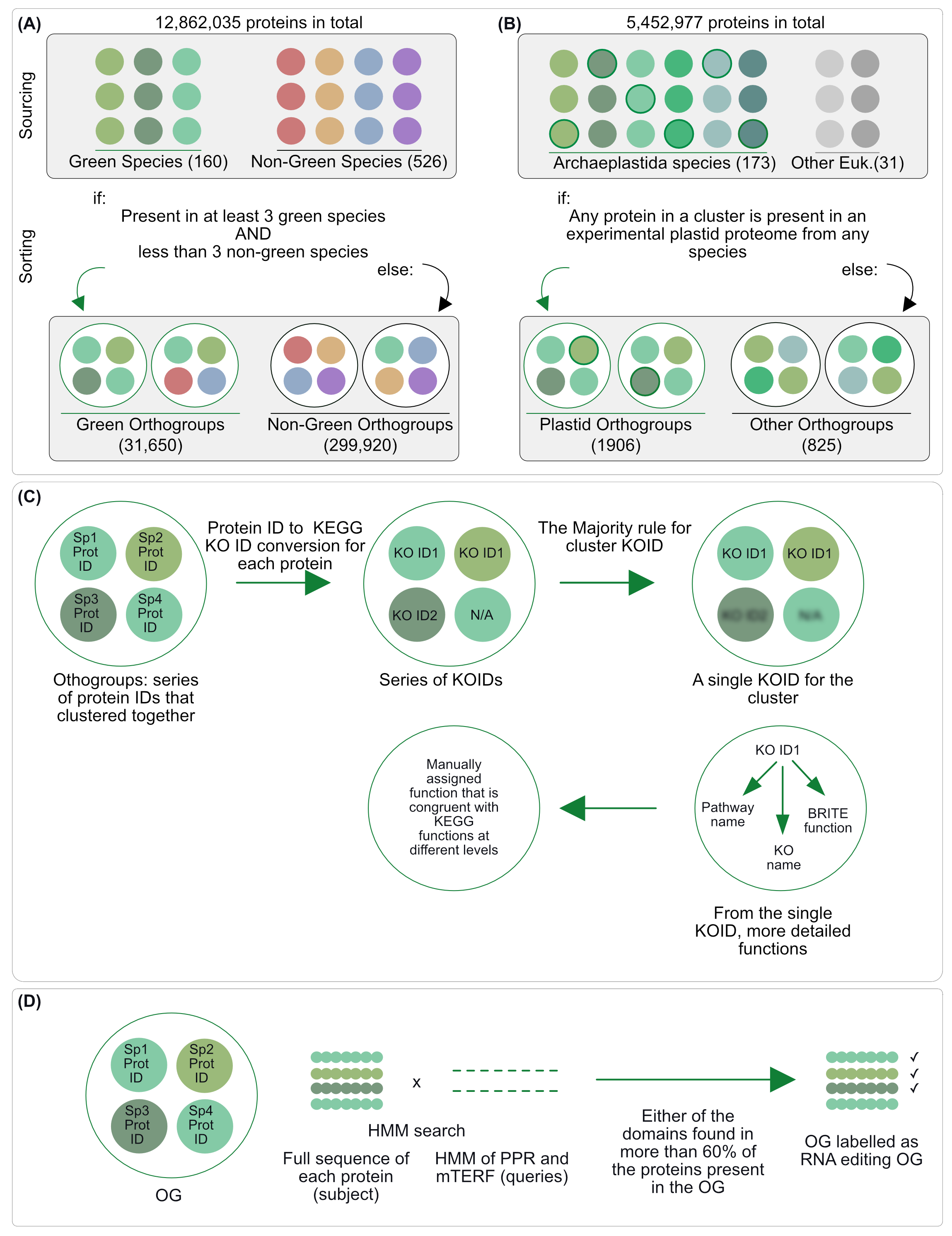

Supplement: S1 Fig — Sorting of (A) the green orthogroups (GOGs) and (B) plastid orthogroups (POGs). In the first step (top boxes), source protein sequences from available species were clustered into protein families. Based on predetermined criteria (between the 2 boxes), the protein clusters were then separated into the clusters of interest (bottom boxes). Mitochondrial orthogroups (MOGs) were sorted the same as (B) and based on their presence in any experimental mitochondrial proteome. (C) For the functional annotations (for GOGs, POGs, and MOGs), KEGG KOID were translated from ProteinID of each protein present in each cluster and from across species. For species outside of the KEGG database (or some proteins within the KEGG database), no KOIDs are available, they are indicated by “N/A.” The most frequent KOID within a cluster (e.g., KEGG ID 1 in the second circle) was assigned as the KOID for the entire cluster. (D) Ogs were sorted into PPR or mTERF containing Ogs by using the hidden Markov profiles of these domains as a query against all proteins in a given OG. If more than 60% of individual proteins inside an OG contained these domains, we labeled it as RNA editing domain. (TIFF) [file pbio.3002608.s001.tiff]

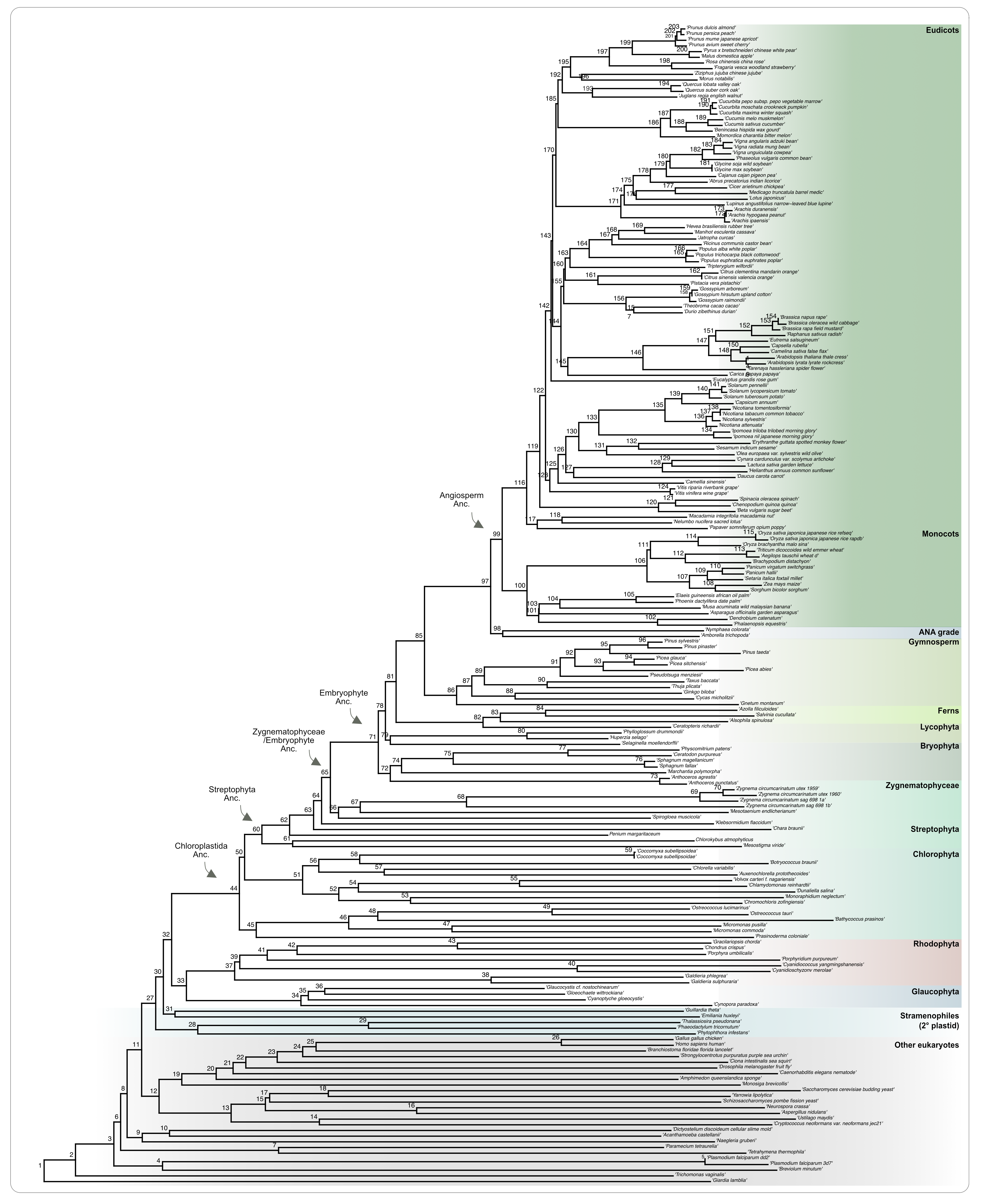

Supplement: S2 Fig — Inferred phylogeny of 204 eukaryotes, with major groups and ancestors of Archaeplastida indicated. The underlying data of this figure can be found at https://zenodo.org/records/10855592. (TIFF) [file pbio.3002608.s002.tiff]

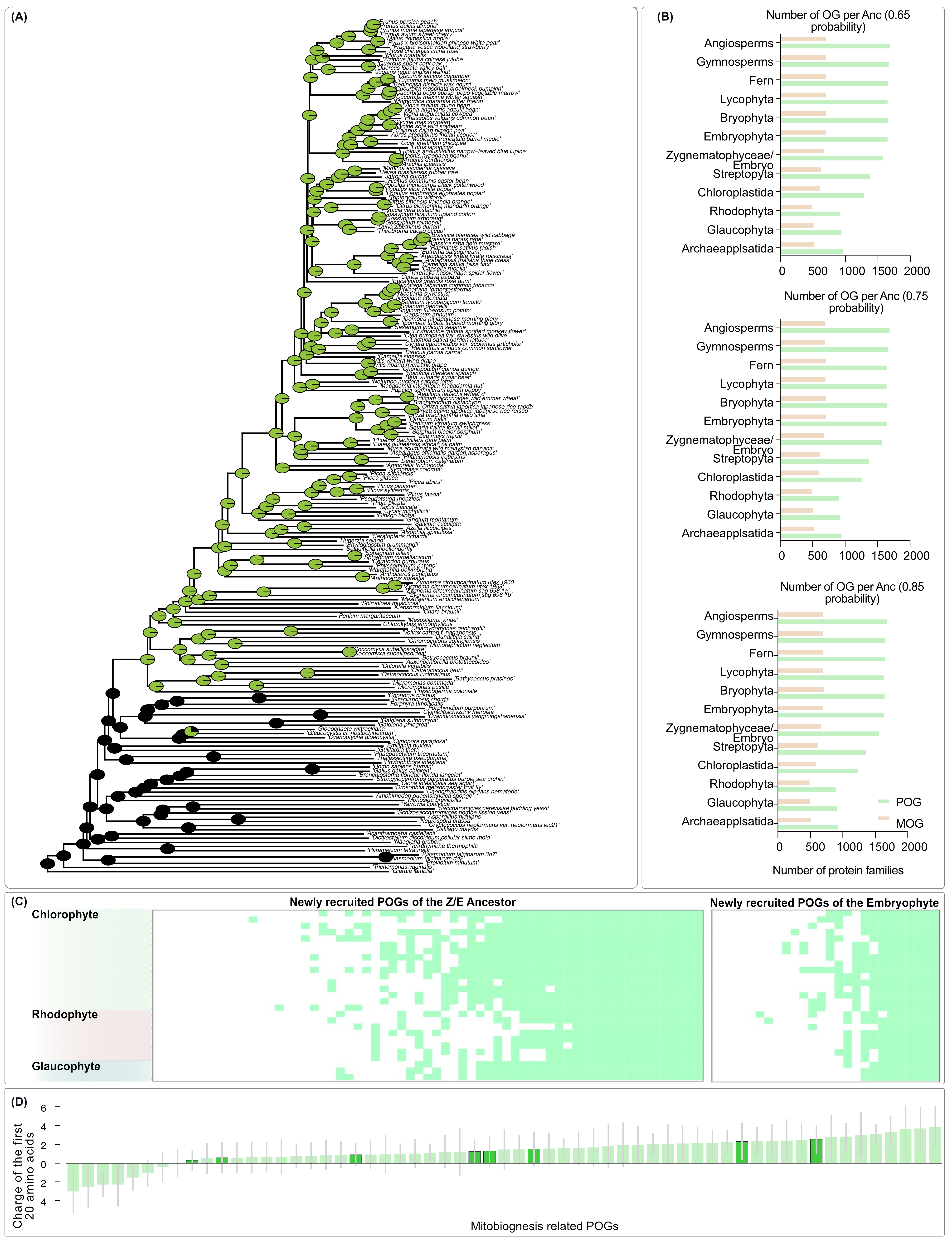

Supplement: S3 Fig — Validation of ancestor state reconstruction (ASR) approach on a control protein family of rbcS (A). Number of POGs and MOGs gained by major ancestors, as per probability threshold of inclusion 0.65, 0.75, and 0.85 (B). Hidden Markov model-based validation of newly gained POGs of Z/E and Embryophyte ancestor (C). Charge of the first 20 amino acids across mitochondrial biogenesis related POGs (D), with mTERF containing POGs present also in MOG shown in a darker shade. The underlying data of this figure can be found at https://zenodo.org/records/10855592. (TIFF) [file pbio.3002608.s003.tiff]

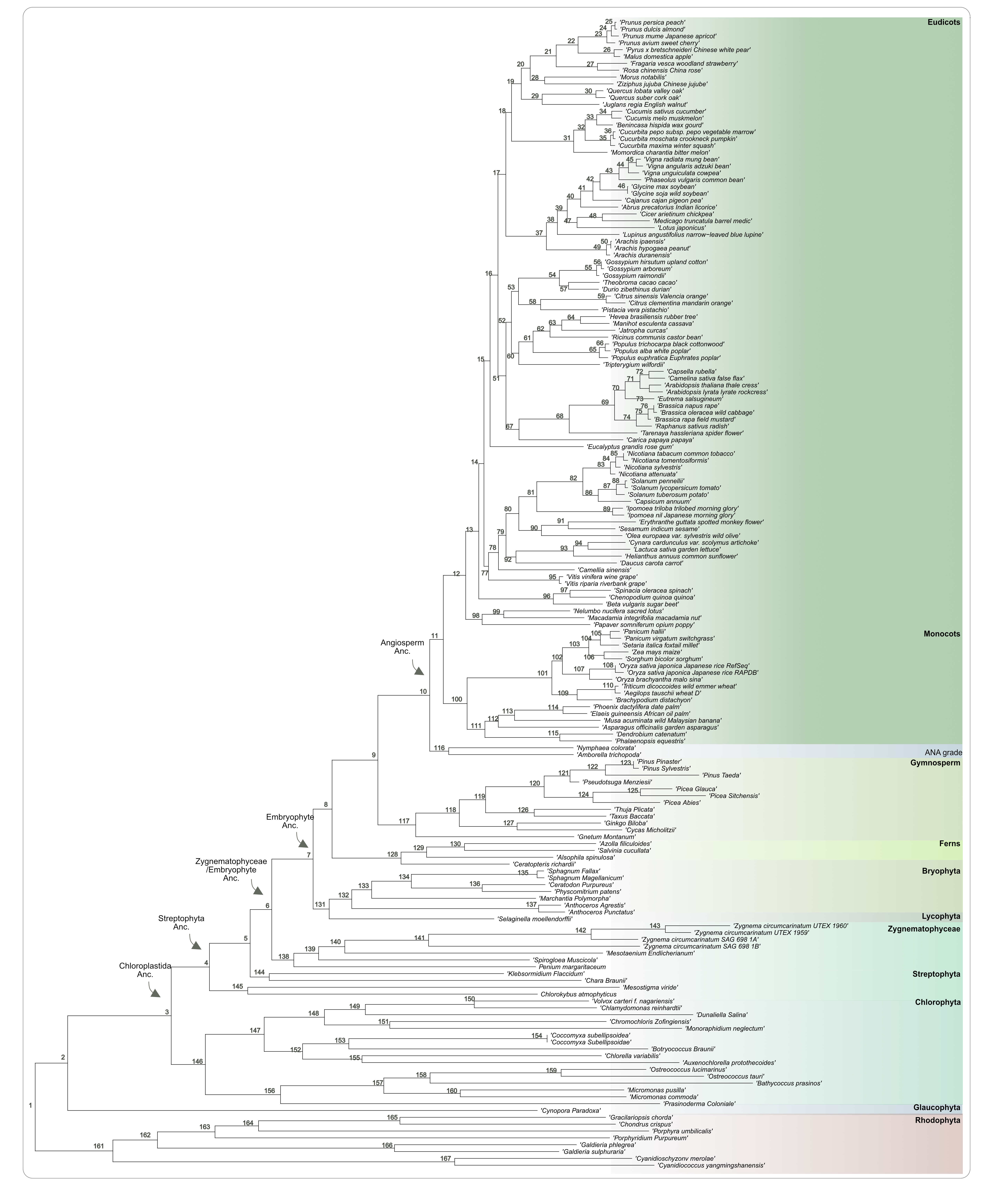

Supplement: S4 Fig — Inferred phylogeny of Archaeplastida (rhodophytes as the sister lineage to all others) with major ancestor nodes indicated with the arrows and major groups highlighted by labels on the right. The underlying data of this figure can be found at https://zenodo.org/records/10855592. (TIFF) [file pbio.3002608.s004.tiff]

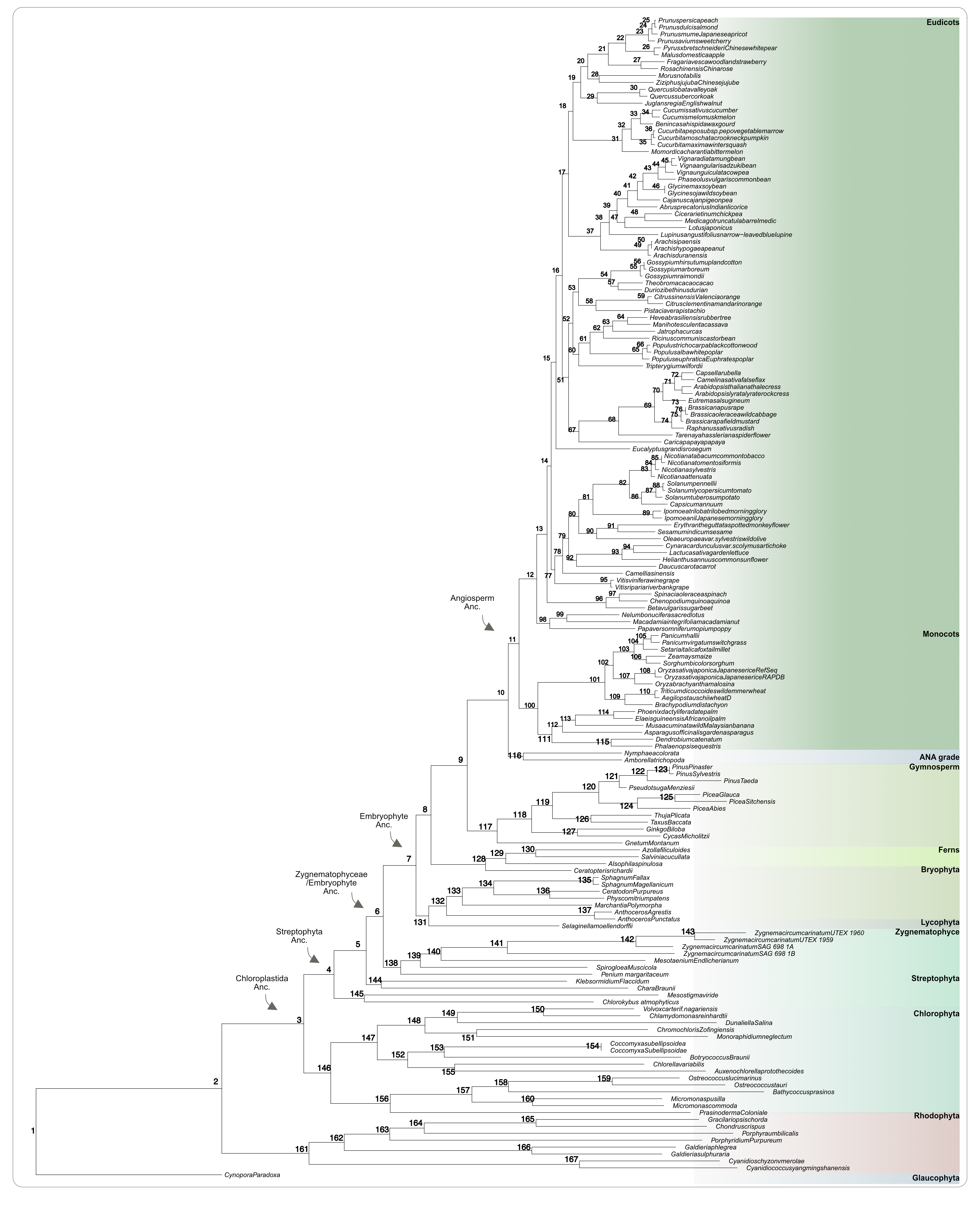

Supplement: S5 Fig — Inferred phylogeny of Archaeplastida (glaucophytes as the sister lineage to all others) with major ancestor nodes indicated with the arrows and major groups highlighted and labeled on the right side. The underlying data of this figure can be found at https://zenodo.org/records/10855592. (TIFF) [file pbio.3002608.s005.tiff]

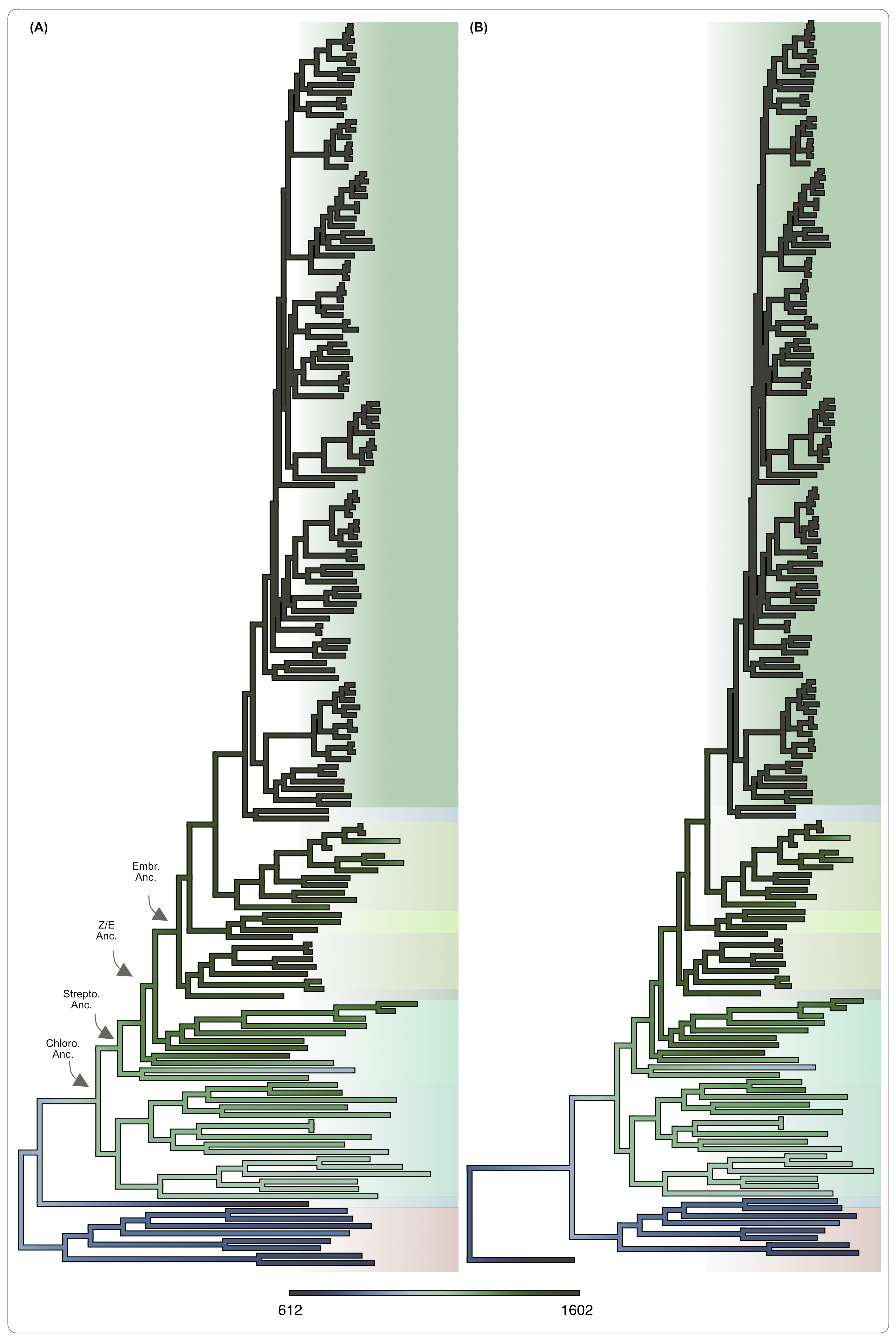

Supplement: S6 Fig — Evolution of plastid orthogroup numbers across the Archaeplastida on a phylogeny with Rhodophyta (A) and Glaucophyta (B) as a basal branch. The underlying data of this figure can be found at https://zenodo.org/records/10855592. (TIFF) [file pbio.3002608.s006.tiff]

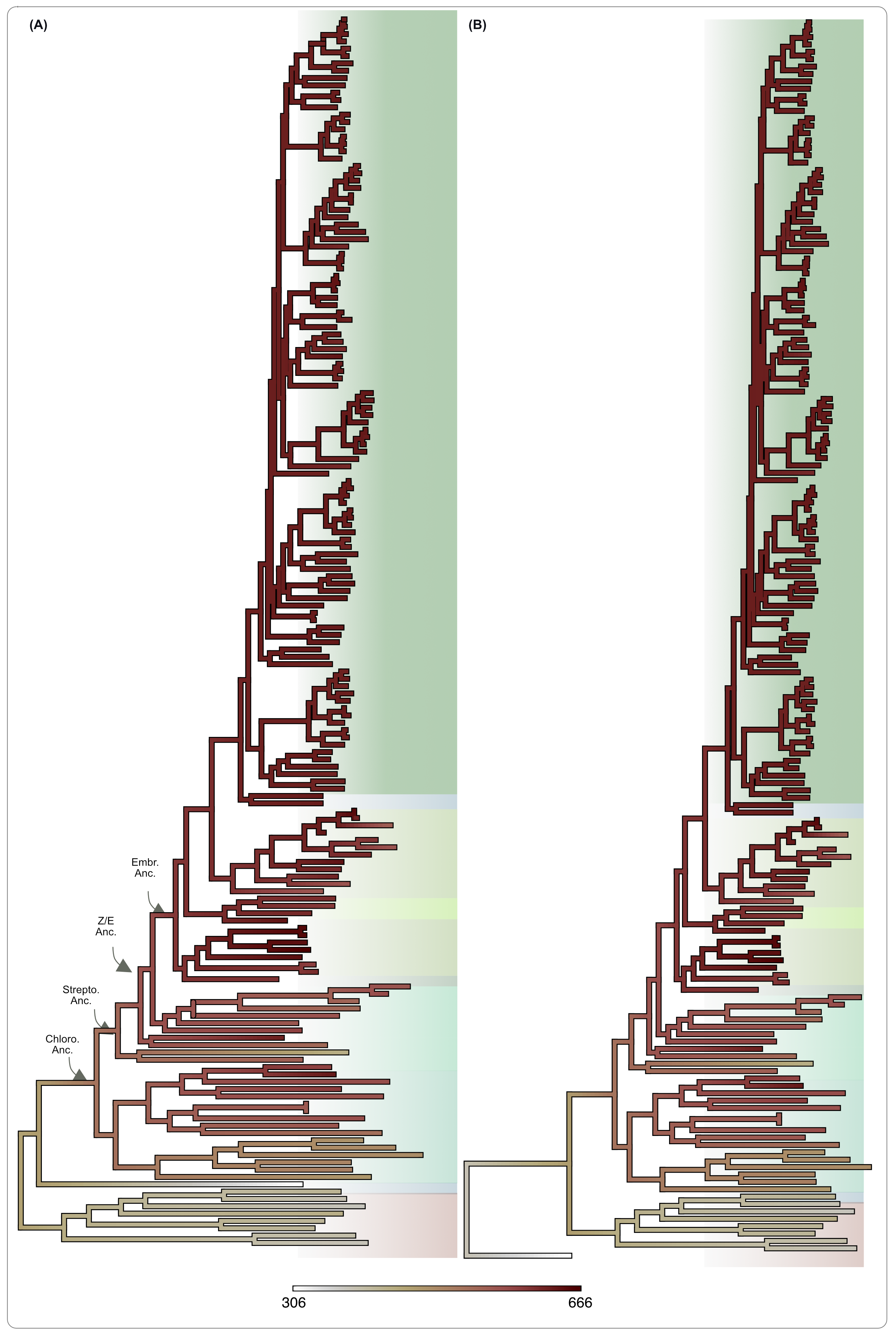

Supplement: S7 Fig — Evolution of Mitochondrial orthogroup numbers across the Archaeplastida on a phylogeny with Rhodophyta (A) and Glaucophyta (B) as a basal branch. The underlying data of this figure can be found at https://zenodo.org/records/10855592. (TIFF) [file pbio.3002608.s007.tiff]

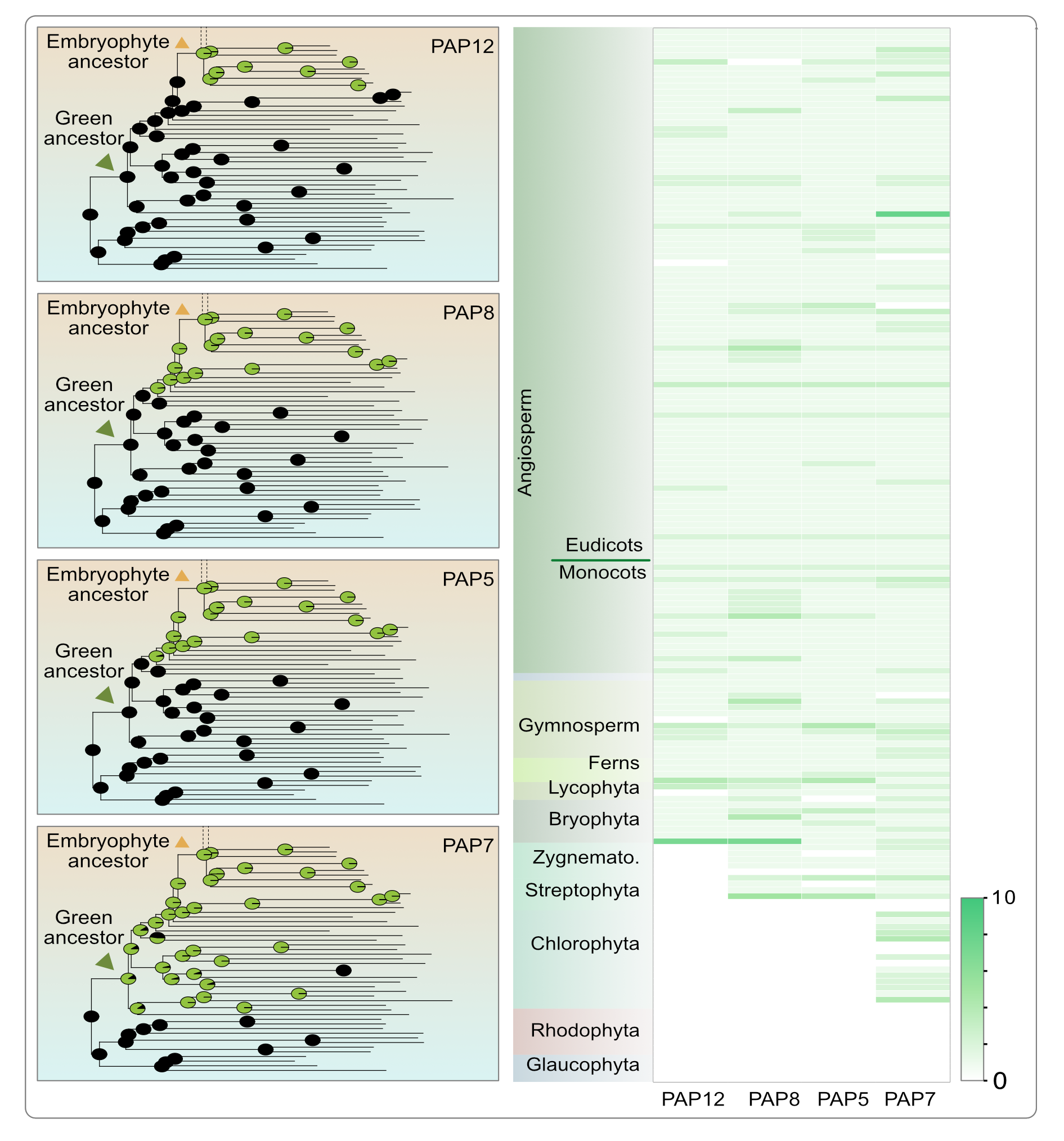

Supplement: S8 Fig — Ancestor state reconstruction (ASR) (A) and gene copy numbers (B) for selected plastid encoded RNA polymerase interacting proteins (PAPs). The pie charts at each node represent the probability of presence (green) or absence (black) of a protein family in that node. The underlying data of this figure can be found at https://zenodo.org/records/10855592. (TIFF) [file pbio.3002608.s008.tiff]

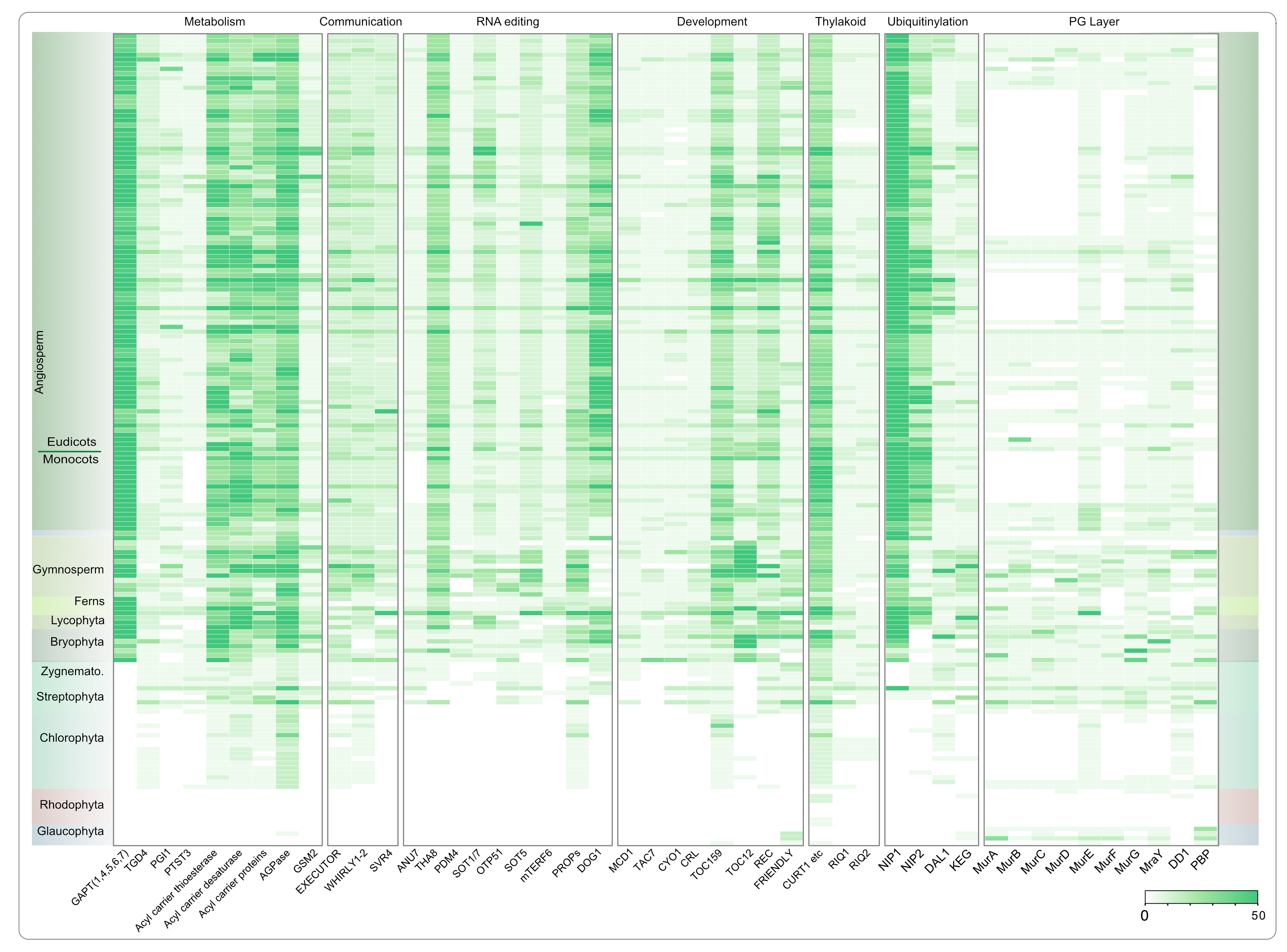

Supplement: S9 Fig — The underlying data of this figure can be found at https://zenodo.org/records/10855592. (TIFF) [file pbio.3002608.s009.tiff]
