# Supplementary material for: A molecular atlas of plastid and mitochondrial proteins reveals organellar remodeling during plant evolutionary transitions from algae to angiosperms
Source: PLoS Biol. 2024 May 7;22(5):e3002608. doi: 10.1371/journal.pbio.3002608 (PMC11135702; doi:10.1371/journal.pbio.3002608)
Supplement: Attachment — Submitted filename: Response to Reviewers.docx [file pbio.3002608.s013.docx]

Dear Dr. Roberts,

we thank the three reviewers for the time they invested in evaluating our study (PBIOLOGY-D-23-02419R1) and your careful and perceptive editorial summary. We have carefully revised the manuscript and have addressed all concerns raised. As requested, the entire analysis concerning organelle proteins was repeated with transcriptome data on Glaucophyta and Lycophyta and an additional glaucophyte plastid proteome, along with the controls and additional analysis suggested (updated are Fig. 2-5, Fig. S1-3, Fig. S8-10). The broad patterns of organelle proteome expansion remain unchanged, nevertheless, conclusions are drawn more carefully and the wording was changed where needed. In addition, thanks to the feedback on bioRxiv preprint, we also revised the RNA editing section substantially. We hope the revision satisfies the reviewers requests and that our manuscript can now move on in the publication process.

Find our response to each reviewer, point-by-point, below. The line numbers refer to the ‘Revised Article with Changes Highlighted’ file.

**Response to R1**

This is an interesting, and ambitious attempt to reconstruct the organelle proteome innovations associated with the terrestrialisation of land plants. The authors show in particular the large number of key chloroplast and mitochondrial proteins associated with modern plants that originate within the Viridiplantae common ancestor, and also the zygnematophyte/ embryophyte common ancestor. The homology analyses are not particularly elaborate but are performed consistently across a large dataset of proteins and species, and can provide us with a lot of information about the stepwise accumulation of « plant-associated » organelle proteins.

**[AU]:** We sincerely thank R1 for his/her time. Addressing all concerns raised has significantly improved our initial submission.

My concerns are not so much to do with the methodology of the paper, or the results obtained, but unfortunately the conceptualisation and questions posed. These do not invalidate the results already obtained but would require substantial additional analysis to address well, or at the least a significant toning down of the conclusions within the paper. In brief: Organelle-associated proteins are defined by occurrence in at least one of four experimentally validated proteomes, three from plants and one from a green alga. This is reasonable (in the absence of validated plastid proteomes from red algae or glaucophytes) but naturally biases our understanding to what makes up a chloroplast to what is found in plants. Thus, the authors should be very cautious to suggest a gain in organelle proteome complexity in the land plant lineage: perhaps they just detect a greater proportion of the plastid proteomes due to closer homology to the experimental datasets used.

**[AU]:** Right, we have addressed this by both carefully checking our text and toning down where required, and by adding more data and re-running our entire analysis. We now consistently also screened non-archaeaplastidal eukaryotes (including some with secondary endosymbionts) to better highlight the changes that culminated with primary and secondary plastid gains. To this end, we conducted an analysis of 173 archaeaplastida, 7 secondary photosynthetic eukaryotes, and 24 non-photosynthetic eukaryotes (covering the available diversity of eukaryotes from three genome databases), which undergirds our main results.

We also note that an increase in proteome complexity is documented by experimental data (e.g. 875 in the alga *Chlamydomonas* vs 1600 in *Arabidopsis*), and a subset of individual proteins that are inferred to be absent in algae are corroborated by the absence of the phenotypic manifestations of those proteins, e.g. polyplastidy. We narrow down the discussion on those (and related pathways), from the hundreds of inferred gains, because for many protein families it remains inferred only and dedicated studies are needed. Our aim was still to picture broader evolutionary patterns, while also suggesting candidates for downstream analyses.

Lastly, we used hidden Markov models (more suitable to capture distant homologues, see Fig. S3C and lines ##170-172) to verify that more than half of the new organelle recruits during the time of terrestrialization (Z/E ancestor and embryophyte ancestor) are absent in chlorophyte algae, rhodophytea and glaucophytes. Where matches are found, they can be explained by a rather generous homology search that is HMM to a degree. The observed pattern does also not change (in any significant manner), with the added plastid proteome of the glaucophyte *C. paradoxa* (and now a number of glaucophyte transcriptomes, Fig 2-3, Fig S2B, Fig. S3).

An equally valid question for which good experimental data do exist: what mitochondrial proteins identified in non-photosynthetic eukaryotes are absent from archaeplastida? Can the authors infer any trends concerning the loss of mitochondrial functions as a result of the acquisition of photosynthesis? Provided the non-green species in the dataset include ones with experimentally resolved mitoproteomes, this information should already exist in the authors' data.

**[AU]:** Excellent question and something we did not consider. Based on human mitochondrial proteome data we identified 4,403 MOGs. More than half were present only in Metazoa, the rest of the eukaryotes (including plants) range from 1,000-1,500 MOGs. *Prima facie*, plants did not lose significant MOGs, while metazoan appear to have gained a lot. However, any statement we would deem print ready and worthy would require a much deeper and dedicated analysis, considering also sequence divergence of the last two billion years and supergroup specific trajectories mitochondria experienced (think hydrogenosomes and mitosomes, absent from plants). In line with our current article and title (one super group, and a focus on half a billion years), we prefer to focus on mitochondria in eukaryotes with plastids.

Similarly, protein localisation can change over evolutionary timeframes and with evolutionary distance: dual-targeted proteins and protein families independently recruited to plastids and mitochondria (e.g., PPRs) are great examples. In the absence of either rigorous in silico or experimental localisations, the authors should not imply that evolutionary homology to one known plastid protein equates to plastid-associated functions across an entire protein orthogroup. This could be probed by custom in silico targeting predictors (e.g., PredAlgo), and/ or identification of coexpression trends in species for which extensive transcriptome data exist (i.e., genes coexpressed with others of known photosynthetic function might be expected to code for plastidial proteins).

**[AU]:** Point taken, with a spot-on example. A homology of sequence does not imply the same localization and we need to be as careful here as we are on the domain analysis, not simply trusting orthofinder per se (I quote from our text: ”*A closer look revealed that most of the algal homologues lacked PPR and mTERF domains and they are hence unlikely true orthologues. More generally, this shows that any detailed interpretation regarding an inferred orthogroup’s function should be supported by screening for functionally relevant domains*.”). On targeting prediction: we indeed intended to do exactly this, but noticed early on (a year ago) how unreliable the tools unfortunately were outside their training species (angiosperms). In fact, we tested this in such detail that it justified an independent publication, which is available as a preprint (https://doi.org/10.1101/2023.12.18.572050). The co-expression pattern seemed a reasonable approach, but showed that plastid localized proteins are distributed across more than a dozen co-expression clusters for *C. reinhardtii* for example (Zone *et al*., 2015; 10.1105/tpc.15.00498).

The clustering approach, while it sets thresholds reasonable for the detection of proteins shared across multiple green species, does not apply robust criteria for conservation of proteins in individual sub-lineages, and I am concerned that contamination or HGT into individual libraries considered might lead to an overestimation of the origin points. Beyond the subsampling approaches used, have the authors tried playing with the species thresholds (e.g., two green algae minimum required to assign Viridiplantae origin) to identify origin nodes?

**[AU]:** Ancestral nodes of interest, around the water to land transition (e.g. zygnematophyceae and embryophyte) are supported by over a dozen of genomes and robust with respect to contamination. Nodes such as at the ancestor of Marchantia and Physcomitrium (where sample sizes are a few) may be prone to contamination, which needs to be respected and therefore not discussed in the manuscript. As for HGT, it has been speculated that it contributed to the trajectory from water to land (https://doi.org/10.1016/j.molp.2022.02.001), an aspect we do not think deserves more attention in our study. The origin nodes for POGs and MOGs are supported by an additional ancestral state analysis, where, if a given ancestor has a probability of presence > 0.75 for a protein family and no preceding ancestors on the phylogeny has that protein family, we consider the given ancestor as origin node. We now also provide a sliding window threshold of 0.65 and 0.85 (Fig. S3), which does not change the broad pattern or numbers by more than 5%. Considering that probability of presence > 0.75 will at least require two species (generally, many more) stemming from that ancestor, it already accounts for a minimum species threshold, especially for the major ancestors.

In the opposite direction, while the authors have prioritised high-quality reference genomes, the paucity of sampling for specific groups (i.e., one lycophyte, glaucophyte, and palmophyllophyte species) are likely to underestimate the organelle protein complexity associated with these lineages, due to library incompletion and species-specific secondary loss. Particular concern are the glaucophytes given their relevance to assigning protein innovations found in the Viridiplantae common ancestor and secondary losses in Rhodophyta : here I feel the authors should judiciously augment their sampling with the more complete reference transcriptomes available for some species (e.g., Gloeochaete wittrockiana 1Kp and MMETSP transcriptomes, Cyanoptyche gloeocystis and Glaucocystis nostochinearum 1Kp transcriptomes).

**[AU]:** As suggested, we ran the analysis again including the glaucophyte transcriptomes and two dozen other eukaryotes. The eukaryotic tree was rooted using minimal ancestral deviation (10.1038/s41559-017-0193) and while Archaeplastida separated as a group, the glaucophyte and rhodophyte were sisters to each other. The broad patterns from the chloroplastidal ancestor onwards (also the focus of our article) did not change (Fig S3), even after including a glaucophyte plastid proteome.

Minor points :

Line 34 : « chlorophytes, streptophytes and the prasinodermophytes that are thought to be the sister lineage to the two former » - is this universally agreed ? It is contradicted by the topologies shown in figs. S2 and S3

**[AU]:** Well, we prefer to stay out of a crossfire here, but indeed also noticed this. As of now there is only that isolated and original report claims prasinodermatophyte to be a sister taxa to former two. One thousand transcriptome initiative found it to be unstable branches. In our analysis, a species tree constructed by Orthofinder (rooted using STRIDE), the same tree rerooted using Minimal ancestral deviation, and an Archaeplastida only tree manually rooted to get rhodophyte and glaucophyte at the base – these all group Prasinoderma as sister to Micromonas (Fig S2-4).

Line 35: it would be fair to note that some terrestrial chlorophytes (e.g., lichen symbionts) exist and are ecologically relevant.

[AU]: While lichens (a symbiosis between fungi and mostly chlorophyte algae) are considered by some “the second terrestrialization by chlorophyte algae” [Asplund and Wardle 2017], we struggle a bit identifying the right spot (and reason) for squeezing this in, because our first paragraph does not state the contrary and we find it problematic to talk about lichens here and so early on the rest of the manuscript is not on lichens and the entire flow then seems problematic to us authors.

Line 38: similarly, polyplastidy is not exclusively the domain of terrestrial embryophytes, although it is frequently observed in multicellular and sessile groups (red algae, giant kelps)
**[AU]:** True and as mentioned in the original article (de Vries and Gould 2018). This is now also better phrased in the revision.

Line 53: as a discrete (targeting) question per the above, it would be interesting to probe the embryophyte-associated orthogroups for peroxisomal targeting motifs, which may be comparatively easier to spot than cTPs or mTPs. Similarly, what proportion of the embryophyte-associated orthogroups have signal peptide positive predictions? Evolution of the plastid has big impacts on the endomembrane system (dual-targeted proteins, rerouting of plastid glycoproteins through the Golgi, etc).

**[AU]:** This is a curiosity we resonate with, especially considering the proposed evolutionary origin of peroxisomes from mitochondria-derived vesicles (Gould et al. 2016.) As much as we would like to probe this, we do not find the current algorithms reliable (see the mentioned preprint).

Line 115: by curiosity, have the authors tried to plot deviations in the ratio of POGs to MOGs recovered in individual species, to identify lineages with possible secondary reductions in plastid function ? I do not see any secondarily non-photosynthetic species in the authors' dataset but the inclusion of facultative mixotrophs (Auxenochlorella protothecoides, Chlamydomonas reinhardtii) could be used to probe if the loss of mixotrophy causes a simplification or elaboration of plastid metabolism.

**[AU]:** We did check and the ratio of POGs to MOGs from the green algae to angiosperms is around 2.2-2.4, which is lower than glaucophytes and rhodophytes (around 1.8). The ratio of protein per POG to protein per MOG also remains comparable across species. There is nothing truly obvious that we deem worthy of explicitly mentioning based on this limited analysis. With respect to the topic (e.g. terrestrialization) a focus is more on gains than losses. And bringing into play species such as Polytoma or Polytomella (secondarily heterotrophic, photosynthesis loss) would not add much apart from the obvious and which has been published (e.g. Smith and Lee 2014; Figueroa-Martinez, et al. 2017). Also note that MOG changes in all of eukaryotes is a much more involved issue. It touches upon plotting the origin of the LECA and the diversification of the (8-10?) major eukaryotic supergroups. We would find us discussing what happened between excavates and opisthokonts, and lose focus of ‘algae vs. angiosperms’.

Line 121: the mitochondrial biogenesis proteins identified as POGs would be interesting to probe at the level of protein targeting. Can the authors say anything about transit peptide length or charge, consistent with dual or ambiguous targeting, in any of these orthogroups?

**[AU]:** The NTS show mean charge of 0-2, and would likely fit a profile for dual targeting (Fig S3D, lines 134-136), although this has to be verified experimentally.

Lines 168-172: PPR proteins are certainly important for RNA processing in plants, but may have more limited roles in other lineages (I only know, for example, of one experimentally validated PPR in apicomplexans). Did the authors look at the distribution of OPRs, TPRs, or other adaptor proteins known to function in RNA processing in other algal groups? What about more broadly the distribution and diversification of organelle RNA nucleases, which are of equal importance for regulating non-coding transcript accumulation in plant plastids and mitochondria?

**[AU]:** These are both fair questions, but considering for instance the hundreds of RNA editing sites and dozens of organelle proteins involved (<https://doi.org/10.1093/plcell/koad049>), this is more of a stand-alone study. We are afraid it would inflate the study at hand too much.

Line 184: can the authors say anything about the predicted targeting sequences associated with the mTERF OGs?

**[AU]:** Their charge and number of phosphorylatable amino acids do seem to fit a profile for dual targeting (Fig S3D) and several are identified in MOGs and POGs both, making them more likely dually localized and one of them (mTERF6) is experimentally verified as dual targeted.

RNA processing section: it would be good to discuss nucleus-encoded RNA polymerases as a known « control » for a green-specific plastid innovation.

**[AU]:** Nuclear-encoded RNA polymerases (NEPs) are encoded by three copies: AT2G24120 (plastid), AT1G68990 (mito), AT5G15700(dual) (cluster OG0001509). Sigma factors (AT1G08540, AT1G64860, AT3G53920, AT2G36990, AT5G13730) for the NEPs also cluster together (OG0001509). Homologues of both these protein families are found in rhodo- and glaucophytes, likely due to a generally conserved nature of RNA polymerases. However, some components of plastid-encoded polymerases (PEPs) are indeed found in the nucleus across the green lineage; we capture them as such: AT1G21600 (PAP8) is unique to zygno onwards, PAP5 (streptophyte onwards) AT4G20130 (PAP7) chloroplastida, PAP12 (Emryophyte ancestor) (Fig S8).

Biochemistry section: a prominent difference between green and non-green plastids is the use of a plastid-encoded form ID rubisco in rhodophytes and glaucophytes. I would presume that the nucleus-encoded small subunit IB rubisco would be detected as a «Viridiplantae-associated gain» in the authors data, which while erroneous would be a good control for the robustness of the orthogroup reconstruction pipeline. I wonder also if there are any plastid-encoded functions in the red lineage that are either detected as Viridiplantae-associated gains (i.e., green-specific EGTs) or indeed are universally absent from Viridiplantae nuclear and organelle genomes.

**[AU]:** Thanks for suggesting a useful control, we now open our ASR section with this. RBCS1A (At1g67090), RBCS1B (At5g38430), RBCS2B (At5g38420), and RBCS3B (At5g38410), all cluster together, as orthogroup OG0001022, which is present across all Viridiplantae and as per our ASR pipeline and predetermined threshold of 0.75 (probability of presence), it evolved in the Viridiplantae ancestor Fig S3A, lines 155-160). As for the red lineage, Rhodoyphte plastids in addition to what is found in green plastid genomes encode extra proteins/enzymes of carbon assimilation, pigment biosynthesis and a lot of ribosomal genes. The latter will not be found as gains as they are clustered with the ribosomal OGs in general; pigment biosynthesis in reds is different, too, considering phycobiliproteins that greens no longer have (and expanded LHC family and that makes it tough. Carbon assimilation, too, is a tough one considering starch metabolism in plastids (red) versus cytosol (green). Would maybe require a detailed “gene-by-gene” analysis that we agree is maybe worthy of doing, but beyond the scope of our analysis on “everything”.

Plastid biogenesis section: where does peptidoglycan biosynthesis fall in the OG data ? This would be a plastid OG that should be shared between glaucophytes, KCC streptophytes, and bryophytes to the exclusion of embryophytes and (presumably) chlorophyte lineages.

**[AU]:** The inferred toolkit for peptidoglycan layer biosynthesis is indeed present in streptophyte and bryophoytes, however, also in many other chloroplastida species (added in Fig. S9). Glaucophyta appear to encode most of the proteins (S9) and serve as an internal control where peptidoglycan layer is experimentally verified (<https://doi.org/10.1508/cytologia.81.357>). The patchy presence of this inferred toolkit across the green lineage (Fig. S9) is intriguing and begs experimental exploration in angiosperms. See also a separate article by our group (https://doi.org/10.1007/s00709-023-01886-y) and others (https://www.mdpi.com/2073-4409/12/4/563).

Lines 279-285 : do any of these TOC proteins show tissue-specific paralogues in embryophytes? Can the authors differentiate between possible « root » and « leaf »-associated isoforms?

**AU]:** – As per *Arabidopsis* transcriptome data (TAIR) TOC159 appears to be overexpressed in leaves and TOC120 in roots and flowers (mentioned now in discussion). In general interesting, yet would likely also require an analysis by itself and like ours, but on a single species and its tissues, asking for “expression gains or silencing”.

**Response to R2**

The authors present a large analysis of gene content in diverse organisms, emphasizing plastid-associated genes. There seems to be supplementary data that I did not have access to, so I do not have an exhaustive list of the taxa that were analyzed, but I can estimate the taxonomic scope from the phylogenetic trees presented. Overall, the analysis seems to have been carefully conducted.

**[AU]:** We thank R2 for his/her willingness to review our work. We apologize that the reviewer was not able to access to the supplementary data we had submitted (a taxonomic table was part of our original submission on bioxRiv; table S1A). Next to this revision, we have also updated our bioxRiv preprint that includes all large supplementary tables. DOI: https://doi.org/10.1101/2023.09.01.555919.

The writing itself is sometimes awkward or grammatically incorrect; there are a number of sentence fragments, malformed clauses, peculiar word choices, and ambiguous turns of phrase. Sometimes this is merely distracting, but at times the intended meaning is genuinely difficult to infer. It appears that the first author is a junior scientist and is still developing their writing. That said, there are a few things that need to be cleaned up. Latin binomials (e.g. genus and species) need to be italicized, but family and above are not italicized. In many cases the binomials are not italicized.

**[AU]:** We proofread the entire manuscript keeping this comment in mind and fixed the text were needed accordingly.

I personally found some of the colonial language jarring. Literally "aboriginal" means "from the beginning" but in context, I found the implication could be read as primitive which is possibly harmful. "Conquest of the land" implies both a volition in evolution and has an inherent violence.

**[AU]:** That is a first for us and something we did not anticipate considering our very own backgrounds. The word aboriginal was used in the very same sense as it was used by Woese and Fox with respect to their seminal paper(s) on the 3 domains of life; sort of paying respect to their work. Some other words and phrasing might come across colonial, but is not always easy to “fix” considering the evolutionary process and nature’s own action. Plants settle in new habitats, territorial animals defend their ground and young bears, wolves, foxes, etc. go out and literally overpower their opponents in a new habitat aggressively. It is very human that we project our problematic behavior and history as a species onto the plant and animal world, but there are limits and nature remains nature. What else would you call a “bacterial colony”? This is not meant as an excuse, but for context. We very much respect R2s concern and did what we could to rephrase our text.

Importantly, the manuscript also includes language that is over-interpreting what is justified by the data. Wording is often credulous when there is substantial uncertainty. At times this extends to statements that are not supported by the underlying facts. For example, regarding mono- vs poly-plastidy, the description in the manuscript does not reflect the taxonomic distribution of polyplastidy from De Vries & Gould 2018. For example, when describing polyplastidy authors ignore the polyplastidy found in the stoneworts (Chara, Nitella, etc), as well as in Bryopsidales and Dasycladales, none of which is sister to Embryophytes. It is true that most green algal lineages are monoplastidic, but the manuscript would be stronger if it didn't sweep the exceptions under the rug.

**[AU]:** Some of the vocabulary used here is quite harsh and not justified. Nothing is swept under the rug, the opposite is true. The very manuscript from 2018 that R2 refers to has multiple question marks in the main figure 1, e.g. where morphological data is uncertain, while Chara and Bryopsis are evidently labelled as polyplastidic. Another paper we recently published based on ASR touches on polyplastidy also clearly labels Chara as polyplastidic (MacLeod et al. 2022; Fig. 2), and the works discuss it accordingly. We are 100% transparent about this. Never did we say polyplastidy is unique to embryophytes, but only a trait associated with this lineage’s origin and macroscopic phenotypes in general. We have made this clear in the revised text and could not identify any other cases, where our text could be “credulous”. We make predictions on the basis of bioinformatics – and yes, many thereof – that can now be tested in the lab. A quintessential component of science, is it not? Should R2 disagree or have evidence for where we are wrong, we would appreciate if he/she could point them out clearly. We are not sweeping exceptions under the rug and why should we in the first place?

Most troubling to me was the discussion of grana, which in the manuscript is described with great confidence as being an Embryophyte trait. A quick review of the ultrastructure literature also shows that while there is a diversity of plastid structure across Chlorophyta, there are well formed grana in most, if not all, streptophytes, and generally similar structures are found in a number of chlorophytes. I may be misunderstanding what the authors mean by the term, but the manuscript as it exists seems to get the phylogenetic distribution of grana completely wrong.

**[AU]:** We need to disagree. First of all, nowhere do we claim “*with great confidence”* that grana stacks are an embryophyte trait and we quote from our paragraph in question: (a) “The evolutionary origins of grana remain elusive, …” (b) “Grana are observed in embryophytes and some Zygnematophyceae (e.g. the *Cosmarium* genus)…”, and (c) “…we speculate that grana originated in streptophytes…”. The latter includes algae and the sentence is nowhere restricted to embryophytes. Second, we cite dedicated literature on the issue that states “Grana, in the form illustrated in Figure 1, appear to be unique to land-plant chloroplasts.” (see e.g. Mullineaux 2005). We note that thylakoid morphology, that is how they align in the stroma, is/was a trait used to distinguish lineages. A lateral heterogeneity of aligned thylakoids (sometimes explicitly exactly two or three from plastid tip to tip) is common, but should not be confused with the characteristic grana stacks of land plants. Our text is more careful on the issue than most published work and our data speaks more towards a grana stack origin in streptophytes than in chlorophytes. We have modified the text slightly to better communicate this.

I don't have time to describe every case where the authors make several very broad claims, but if the manuscript were more circumspect in its assertions it would in fact be a much stronger, not a weaker manuscript. Accurately reflecting where there is uncertainty is valuable. When the authors are speculating and are aware of it, it should be explicit.

**[AU]:** It is difficult to know what R2 might consider a broad claim or to what degree some of these assertations relate to a misreading of published literature or our text (see e.g. the issue on grana stacks or monoplastidy), if they are not explicitly listed. We made sure to only stick our head out, where we trust that our data allows to do so and/or it is beneficial with respect to scientific progress. It’s a fine line, we understand, but modern science suffers from “anxiety issues”. Incremental science is preferred over disruptive science to avoid risk (see e.g. Sutter 2022 or Kozlov 2023 in Nature).

Although the language is rough and at times incorrect, the structure of the manuscript is clear, and the authors make many sophisticated and non-dogmatic assertions from their large analysis. There are places where the manuscript really is a joy to read, or accurately makes difficult inferences. A critical revision focused on closely adhering to the evidence would make it a much more valuable contribution.

L17: "conquer" word choice (WC)

L32: "conquest" WC

L44: "For instance, improving a stress…"

L47: "was part of land plants"

L51: "domination" WC

L68: "at that time." missing citation

L92: The heavy reliance on acronyms (GOGs, POGs, MOGs, etc.) is distracting.

L180: Latin names should be italicized throughout

**[AU]:** Thanks for the positive note on our sophisticated and non-dogmatic assertions. We have checked all listed sections and fixed them (e.g. substituting “conquer” and “conquest”). We don’t understand what citation(s) R2 would expect for genomes and tools that were not published 10 years ago or more? And with respect to the acronyms, what would be an alternative? The categories need names and the change of only the first letter that communicates the category in question seems quite reasonable. Latin names were fixed.

L291: This sentence is an example of how the language in the manuscript is problematic. Ignoring the iffy use of the language of colonization and conquest, the use of the word "propagation" is peculiar (this is normally used for human cultivation of plants), and "…foundation for the concomitant…" is both a malapropism, because "concomitant" means "accompanying" or "traveling as companions," so is conceptually inconsistent with one laying the foundation for the other, but it is also factually incorrect, because there is evidence of animals - chelicerates in particular - having been on land prior to embryophytes.

**[AU]:** The sentence was changed to “The gradual settling of land by a streptophyte alga and the subsequent evolution and spreading of plants (Fig. 6A) was pivotal in the transformation of the terrestrial habitat and it laid the foundation for the concomitant evolution and diversification of animals [1,2].” The word concomitant in the context of the evolutionary time scales and topic we’re dealing with (many millions of years) is not a malapropism. We are more so referring to events such as the origin of collagen, whose biosynthesis depends on O2 levels made possible by plant photosynthesis, and less so about whether a mite might have crawled barren rock before the occurrence of a soil layer from early embryophytes. The vast majority of literature there is on the topic agrees that land animals evolved hand in hand with land plants. If R2 thinks otherwise, he/she might want to discuss so in print.

L293: "this endeavor" and "hereby evolving into" again implies volition

**[AU]:** “Endeavour” was changed to “plant evolution” and the volition one might read into this hereby removed.

L344: This is not correct. A quick review of green algal plastid ultrastructure shows that a number of chlorophytes (e.g., Scenedesmus, Hydrodictyon) have grana. Also: the singular is (to my understanding) "granum", and the plural "grana."

**[AU]:** Please see our earlier reply with respect to grana evolution, morphology and the dedicated papers on the issue we cite. It should have read “grana stacking” not “granal stacking” and was changed accordingly. Thanks.

L356: Good!

**[AU]:** Thanks.

L364: "polyplastidy, evolved in embryophytes" This statement is (probably) true, but the exceptions should be noted - in stoneworts in particular, but also in Bryopsidales and Dasicladales.

**[AU]:** In line with a comment by R1, this is now better phrased.

L385: Word choice: Is "aboriginal" supposed to mean ancient or primitive? possibly "fundamental" or "basal" might be more fitting. The word means native or indigenous which the others are too…

**[AU]:** As mentioned, we used the word in the same sense as e.g. Woese and Fox on their discovery on the 3 domains tree.

**Response to R3**

The authors take advantage of mitochondria and chloroplast experimental proteome data in 4 model species (the freshwater microalgae Chlamydomonas, the moss Physcomitrella , Zea mays and Arabidopsis), to investigate the evolutionary history of the associated gene families. These associated gene families are identified from the genome annotations of 168 phototrophic eukaryotes (Archaeplastida) versus 518 non-phototrophic eukaryotes, and add up to 1961 (chloroplast) and 846 (mitochondrial) gene families. The functional annotation of these gene families enables key insights into the evolutionary history of the proteomes of these organelles during the evolution of Archaeplastida. This manuscript is a pleasure to read, and I really enjoyed the precision and graphic quality of the figures, including the supplementary figures, that are very important to understand the bioinformatic approaches used to build and analyze this massive gene family dataset. I have however several concerns I detail below, I believe should be addressed before I can recommend publication of this manuscript.

**[AU]:** We thank R3 and have worked on every issue raised.

1. The supplemental figures and tables about the methodology are very helpful but the methodological strategy is not described with a sufficient level of details in my opinion. 1a. please provide reference for each method / database used in the methods section

**[AU]:** Done. We have added more references and elaborated Fig. S1C (part on functional annotation).

1b. whenever in house scripts were used to process the data, these should ideally be made available via GitHub or something equivalent, e.g. for the "translation" of functional annotation from protein ID
**[AU]:** Correct, and this has been taken care of (Github repository ‘Molecular-Atlas-of-plant-organelle-evolution’).

1c. "OGs were sorted into PPR or mTERF containing OGs by using the hidden Markov profiles of these domains as a query against all proteins in a given OG. If more than 60% of individual proteins inside an OG contained these domains, we labelled it as RNA editing domain. » Could you please justify the 60% of criteria, is it based on a non-linear distribution of % of hits within gene families?»
**[AU]:** It was an arbitrary albeit predetermined threshold according to common protocols for large scale comparative genomics. The threshold of 60% identified 57 OGs, threshold of 50% also identified 57 OGs. A threshold of 40% and 70% identified 59 and 55 OGs, respectively. We would think it is more critical, whether or not a given species has proteins with these domains as compared to OGs at large.

2. Mitochondrial (MOGs) or Plastidial (POGs) Orthogroups were identified as containing at least one experimentally verified organelle protein from any one of the four experimentally verified organelle proteome of C. reinhardtii , P. patens  , Z. mays,  A. thaliana. This is not necessarily a large enough dataset. Could more information about the comparison of the mt and cp proteome content between these 4 species be provided. This comparative analysis may enable to discuss whether additional proteomes are likely to increase the number of organelle proteins (i.e. many lineage specific proteins)?

**[AU]:** We agree (and in line with R1 in this context) and have added another glaucophyte, which also expands our definition of plastid. We also agree that larger proteome pool would be better, but none is available as of now. Hence, we would also kindly point to the last paragraph before our conclusion, which we start with: “In closing, although experimentally reported plant plastid and mitochondrial proteomes are scarce, we were able to generate a first comprehensive molecular atlas of the changes of plastid and mitochondrial protein families in the evolution of the green lineage. Ancestral state reconstruction (ASR) allows to map the organelle transformations that facilitated the major transitions such as terrestrialization and which will improve with every new proteome that is added.” In addition we added: “Additional proteomes, once available, will likely solidify the general pattern observed and uncover more lineage-specific curiosities.”

3. The title and a few sentences along this manuscript suggest there's a tendency towards "adaptationism", and I believe "the word "adaptation" is used too often (>17 instances) and without evidence, for example: 3a : in Title  « adaptation » -> "evolution" may be more correct, gene family evolution, like the evolution of any genomic trait, is the result of chance and necessity, including adaptations, so I would prefer the use of "evolution" instead of adaptation here, unless the authors can provide some evidence these GF changes are adaptive	?

**[AU]:** Right, we went a little crazy with using the word adaptation and have removed or substituted four cases where needed/appropriate. The title, too, was changed to:” A molecular atlas of plastid and mitochondrial evolution from algae to angiosperms”.

3b- line 59 «mitochondria were continued adaptation" "malate decarboxylation in the C4 photosynthetic pathway [23] - a convergent trait that improves plant photosynthetic efficiency in warm and dry habitats ". It seems that the complete C4 pathway is already encoded in aquatic microalgae see PMID: 18359853 and references therein, so I am unsure it is correct to suggest the associated gene families are associated with adaptation to dry habitats.

**[AU]:** Good point, and we relied maybe too much on citation 23. We now mention the C4 pathway as a preadaptation and mention mitochondrial retrograde signaling under stress as a better example of terrestrial mitochondrial adaptation.

3c- line 70 : please clarify how orthology inference enables to detect adaptations or rephrase.

**[AU]:** Right. ‘adaptations’ was removed.
