# Supplementary material for: A molecular atlas of plastid and mitochondrial proteins reveals organellar remodeling during plant evolutionary transitions from algae to angiosperms
Source: PLoS Biol. 2024 May 7;22(5):e3002608. doi: 10.1371/journal.pbio.3002608 (PMC11135702; doi:10.1371/journal.pbio.3002608)
Supplement: Attachment — Submitted filename: Response to the Reviewers.docx [file pbio.3002608.s014.docx]

**Response to R1**

I am happy with the revisions made by the authors to their manuscript, and their detailed and thoughtful responses to my questions.

**[AU]**: Thank you for the well thought-out comments. Conducting the additional analyses provided food for thought and, needless to say, improved our study substantially.

I have two additional points that I think can be improved in the paper:

1) I still stand by the point that the absence of experimentally validated organelle proteomes for red algae, alongside the lack of reliable targeting predictors for this group, limits our understanding of their composition and complexity. I would like the authors to explicitly discuss this, its implications for their data (e.g., POGs or MOGs unique to red algae, and not found in Chloroplastida) in their Discussion, and it would be helpful to suggest some possible experimental solutions (e.g., LOPIT on model red algal species, or proximity proteomics of transformable species such as Porphyridium with known plastidial markers).

**[AU]**: Agreed and we emphasize this now in more detail (lines 447-462).

2) The complexity of the secondary plastid proteomes in the authors' dataset is likely hugely underestimated, reflecting the evolutionary distance between these and Chloroplastida, and (for targeting predictions) the different pre sequences (e.g. with hydrophobic N-terminal targeting sequences) used to direct proteins to the plastids of these groups. This underestimation is in effect visible in Fig. 2 where the secondary plastid containing groups lack many of the 1y chloroplast-associated POGs, but show equivalent retrieval of MOGs as Archaeplastida. While not the focus of this study, I think the authors could address this readily by computing the overlap between their orthogroups and experimentally validated secondary plastid proteomes, e.g. Euglena (Novak Vanclova et al, 2020) or Phaeodactylum (Huang et al, 2024) to see how many of these secondary plastid-associated proteins escape incorporation into POGs assembled from a Chloroplastida-anchored dataset.

**[AU]**: Thanks for pointing out this pattern that we now discuss in light of niche-specific evolution and sequence divergence between primary and secondary plastids (lines 447-462). The *Euglena* genome was not a part of our study, as it is not available through KEGG. For *Phaeodactylum*, Huang *et al*. identified 2850 proteins in the plastid enriched fraction and, as also stated by Huang *et al*., not all of these are genuine plastid-localized proteins. Further filtering based on the Plant Protein Database (also used by us), shows a substantial contamination from other organelles and leaves them with only 839 plastid proteins, a number not far off from our own (934). Along the same lines, Boucher *et al.,* 2018 experimentally mapped 346 proteins to the *Plasmodium falciparum* dd2 apicoplast, again not far off from our number (329). While a worthy suggestion, it is currently not really easy to address given the current status of secondary genomes and proteomes. We appreciate R1s interest in secondary endosymbiosis (that in particular the senior author shares since his PhD) and agree that this deserves its own study once more data becomes available – ideally through more funding on more exotic species and perfect tools such as LOPIT.

**Response to R2**

The authors seem to have missed the fact that I liked the paper. The changes have conscientiously addressed my primary concerns. Where we disagree is well within the range of authorial discretion. I am now comfortable recommending the paper for publication.

**[AU]**: This has indeed been a pleasant review experience. Thank you, also for carefully addressing some words so carefully; it didn’t hurt to reflect on a potentially problematic wording and it certainly has improved the parts of text in question. We also believe that the disagreement is born out of cultural and philosophical differences worth preserving and we appreciate the authorial discretion that many could learn from.

**Response to R3**

The authors have addressed my concerns and I am happy to recommend this revised version.

**[AU]**: Thank you for balancing our ‘adaptationist’ phrasing here and there.

I still have a (very) minor comment about the use of the word "adaptation"

Line 25 In the abstract, I suggest removing the word 'adaptation' (already used line 17) in "The analyses of gained orthogroups identifies molecular adaptations", as a demonstration that these gains are adaptations may be controversial (while I agree with some examples detailed later in the manuscript). A more correct phrasing of this sentence would be "The analyses of gained orthogroups identifies molecular changes of organelle biology that connect to the diversification of major lineages and facilitated major transitions from chlorophytes en route to the global greening and origin of angiosperms."

**[AU]**: Right, this has now been changed, too.
